# Posterior Segment Involvement and Treatment Outcomes of Closed Globe Injury at Tertiary Eye Care Center: An Observational Study

**DOI:** 10.31729/jnma.9183

**Published:** 2025-08-31

**Authors:** Sundip Dware Chhetri, Raba Thapa, Sudeep Lal Rajbhandari, Nischal Ghimire

**Affiliations:** 1 Tilganga Institute of Ophthalmology, Gaushala, Kathmandu, Nepal

**Keywords:** *closed globe injury*, *ocular trauma*, *posterior segment manifestation*

## Abstract

**Introduction:**

Closed globe injury is a common cause of visual impairment worldwide, affecting both anterior and posterior segments of the eye. This study aimed to determine the posterior segment findings and treatment outcomes in patients with closed globe injury at a tertiary eye care center in Nepal.

**Methods:**

A prospective observational study was conducted on 58 patients with clinically diagnosed closed globe injury at a tertiary eye care center in Kathmandu from February 2020 to July 2021. Ethical approval was obtained from the Institutional Review Committee of the National Academy of Medical Sciences (Ref: 76/2077/078). Detailed demographic, clinical, and ocular examination findings were recorded.

**Results:**

Among the 58 patients, 48 (82.76%) were males and 10 (17.24%) females, aged 18-71 years. Fist injuries were, reported in 19 (32.76%) cases, with the left eye affected in 32 (55.17%) patients. At presentation, 30 (51.72%) had normal vision, while 7 (12.07%) were blind. At 6 months, 46 (79.31%) achieved normal visual acuity, with only 1 (1.72%) remaining blind. Commotio retinae was observed in 27 (46.55%) cases of posterior segment findings. At the 6-month follow-up, 44 (75.86%) had fully recovered, while 14 (24.14%) exhibited posterior segment complications, including 4 (6.90%) with choroidal rupture and 2 (3.45%) with optic disc atrophy.

**Conclusions:**

Closed globe injury significantly contributes to visual disability, with fist injuries and commotio retinae being common. Young adults are predominantly affected. Early diagnosis and management are critical to optimize visual outcomes.

## INTRODUCTION

Ocular trauma is a significant cause of vision loss globally.^1^ Closed globe injuries can affect both anterior (iris, lens)^2^ and posterior segments of the eye, resulting in commotio retinae, Berlin’s edema, macular holes, retinal detachment, choroidal rupture, and optic nerve damage.^3^ The Nepal Blindness Survey reported that 2.4% of blindness was due to eye injuries, with corneal damage as the second most common cause after cataract.^4^ In rural areas, especially among farmers, minor corneal injuries may rapidly worsen, causing ulcers and vision loss.^5^ The Bhaktapur Eye Survey found that 0.7% of individuals had experienced eye trauma.^6^ At a tertiary hospital in Nepal, 98.2% of ocular trauma cases involved closed globe injuries.^7^

Most existing literature is based in developed countries with advanced care. Local data are needed to guide preventive strategies.

This study aims to assess the patterns of posterior segment involvement and treatment outcomes in closed globe injuries at a tertiary eye center in Nepal.

## METHODOLOGY

A hospital-based prospective observational study was performed at Tilganga Institute of Ophthalmology, a tertiary eye care center, Gaushala, Kathmandu, Nepal, from February 2020 to July 2021, following ethical approval from the Institutional Review Board of the National Academy of Medical Sciences (Reference number: 76/2077/078).

This was a duration based study where the total population was taken without calculating sample size with all the consecutive patients with closed globe injuries involving the posterior segment who presented to the Outpatient and Emergency Departments included in the study. Exclusion criteria comprised individuals aged <18 years , those with pre-existing ocular conditions (e.g., diabetic retinopathy, retinal vein occlusion, mature cataract, corneal opacity hindering posterior segment evaluation), prior ocular trauma or surgery, or unwillingness to participate. Informed consent was taken from the eligible patients before involvement in the study. A total of 58 cases of closed globe injury meeting the inclusion criteria were collected over a one-year period.

Detailed clinical history (demographics, symptoms, medical/ ocular history) and ocular examinations were conducted by the principal investigator and co-investigator and the final diagnosis was established. Assessments included Best Corrected Visual Acuity using Snellen’s or Tumbling E charts, extraocular motility, pupillary evaluation including Relative Afferent Pupillary Defect (RAPD) and posterior segment evaluation via slit-lamp biomicroscope (90D lens) and indirect ophthalmoscopy (20D lens). Visual impairment was classified into mild (6/6-6/12), moderate (<6/12-6/60), severe (<6/60-3/60) and blindness (<3/60-No Perception of Light) based on the degree of vision loss.^8^ Data were recorded using a standardized proforma. Patients were followed up at intervals of 1 week, 1 month, 3 months and 6 months.

Data were managed in Microsoft Excel (Microsoft Office 2019). Statistical analyses were performed with IBM SPSS Statistics for Windows, Version 27.0 (IBM Corp., Armonk, NY, USA). The patient’s personal information was kept confidential and data was secured in password protected computer. Non-continuous data were presented as frequency and percentages and continuous data as means and standard deviation.

## RESULTS

A total of 58 patients with closed globe ocular trauma were included in the study. The age of the patients ranged from 18 to 71 years, with a median age of 29 years (Table 1).

There were 48 (82.76%) male patients and 10 (17.24%) female patients. In terms of ethnicity, 36 (62.07%) patients belonged to the Indo-Aryan group and 22 (37.93%) to the Tibeto-Burman group in our study. Regarding occupation, 20 (34.48%) patients were students, followed by 13 (22.41%) individuals in service, 11 (18.97%) in business, 10 (17.24%) in agriculture and 4 (6.90%) were homemakers. There were 2 (3.45%) patients illiterate, 6 (10.34%) patients had primary level education, 32 (55.17%) had secondary education, and 18 (31.03%) had attained higher education.

**Table 1:** Age of patient with closed globe injury (n=58).

Age	n(%)
18-30yrs	35(60.34)
31-40yrs	12(20.69)
41-50yrs	3(5.17)
51-60yrs	6(10.34)
61-70yrs	1(1.72)
>70yrs	1(1.72)

Among the total patients, 19 (32.76%) patients sustained injuries due to physical assault with a fist, followed by 12 (20.69%) patients had trauma with wooden objects and 10 (17.24%) due to falls and 5 (8.62%) due to stones. There were 35 (60.34%) patients who presented to the hospital after 24 hours of injury, while 23 (39.66%) patients presented within 24 hours of injury.

The left eye was involved in 32 (55.17%) cases, while the right eye was involved in 26 (44.83%). At presentation, 54 (93.10%) patients did not exhibit RAPD, while 4 (6.90%) patients had RAPD.

Initial visual acuity assessment showed that 30 (51.72%) patients had normal vision, 6 (10.34%) patients had mild visual impairment, 13 (22.41%) patients had moderate impairment, 2 (3.45%) patients had severe impairment, and 7 (12.07%) patients were blind. The mean visual acuity at presentation was 0.566 ± 0.652 logMAR. At one week follow-up, 42 (72.41%) patients had normal visual acuity, 8 (13.79%) patients had moderate visual impairment, and 8 (13.79%) patients were blind, with a mean visual acuity of 0.44 ± 0.7 logMAR. At one month, 44 (75.86%) patients had normal vision, 3 (5.17%) patients had mild impairment, 3 (5.17%) patients had moderate impairment, 6 (10.34%) patients had severe impairment, and 2 (3.44%) patients remained blind. The mean visual acuity improved to 0.248 ± 0.456 logMAR. At the three-month follow-up, 46 (79.31%) patients had normal vision, 1 (1.72%) patient had mild impairment, 5 (8.62%) patients had moderate impairment, 5 (8.62%) patients had severe impairment, and 1 (1.72%) patient was blind. The mean visual acuity was 0.228 ± 0.456 logMAR. By six months, normal visual acuity was noted in 46 (79.31%) patients, 1 (1.72%) patient had mild impairment, 6 (10.34%) patients had moderate impairment, 4 (6.90%) patients had severe impairment, and 1 (1.72%) patient remained blind. The mean visual acuity at six months was 0.222 ± 0.44 logMAR (Table 2).

**Table 2:** Visual acuity at presentation and after 6 months in patients with closed globe injury (n=58).

Visual Acuity Range	At Presentation n (%)	At 6 Months n (%)
6/6 - 6/12	30 (51.72)	46 (79.31)
<6/12 - 6/18	6 (10.34)	1 (1.72)
<6/18 - 6/60	13 (22.41)	6 (10.34)
<6/60 - 3/60	2 (3.45)	4 (6.90)
<3/60 - No Perception of Light (NPL)	7 (12.07)	1 (1.72)

Retinal attachment was intact in 56 (96.55%) patients, while 2 (3.45%) patients had retinal detachment. 27 (46.55%) patients had isolated commotio retinae in the posterior segment, followed by vitreous hemorrhage in 5 (8.62%) patients, commotio retinae with retinal hemorrhage in 4 (6.90%), patients and isolated retinal hemorrhage in 3 (5.17%) (Table 3).

Regarding treatment, 37 (63.79%) patients had received topical steroids and 9 (15.52%) patients had received combined medical (topical and systemic steroids) and surgical treatment. There were 7 (12.07%) patients who were managed conservatively with observation only, 4 (6.90%) patients had received topical and systemic steroids, and 1 (1.72%) patient had received surgical and topical steroid treatment.

At final follow-up, 44 (75.86%) patients had complete recovery without sequelae. Isolated choroidal rupture was observed in 4 (6.90%) patients, optic disc atrophy in 2 (3.45%) patients, and epiretinal membrane in 2 (3.45%) patients (Table 4).

**Table 3:** Distribution of posterior segment findings in patients with closed globe injury (n=58).

Posterior Segment Findings	n(%)
Commotio Retinae	27 (46.55)
Vitreous Hemorrhage	5 (8.62)
Berlin's Edema	2 (3.45)
Choroidal Rupture	1 (1.72)
Post Traumatic Optic Neuropathy	1 (1.72)
Retinal Hole	1 (1.72)
Commotio Retinae + Retinal Hemorrhage + Vitreous Hemorrhage	1 (1.72)
Retinal Hemorrhage	3 (5.17)
Retinal Hemorrhage + Choroidal Rupture + Macular Hole	1 (1.72)
Commotio Retinae + Berlin's Edema	2 (3.45)
Commotio Retinae + Retinal Hemorrhage	4 (6.90)
Commotio Retinae + Retinal Hemorrhage + Choroidal Hemorrhage	1 (1.72)
Vitreous Hemorrhage + Retinal Detachment + Post Traumatic Optic Neuropathy	1 (1.72)
Peri-Papillary Hemorrhage	1 (1.72)
Vitreous Hemorrhage + Retinal Hemorrhage	1 (1.72)
Commotio Retinae + Berlin's Edema + Retinal Hemorrhage	1 (1.72)
Posttraumatic Optic Neuropathy + Retinal, Macular, Vitreous Hemorrhage	1 (1.72)
Vitreous Hemorrhage + Berlin's Edema + Retinal Hemorrhage	2 (3.45)
Berlin's Edema + Disc, Macular, Retinal, Vitreous Hemorrhage	1 (1.72)
Retinal Detachment + Retinal Hole + Vitreous Hemorrhage	1 (1.72)

**Table 4:** Outcome of patients with closed globe injury following treatment (n=58).

Outcome following treatment	n(%)
Cured	44(75.86)
Optic Disc Atrophy	2(3.45)
Choroidal Rupture + Macular Hole	1(1.72)
Epiretinal Membrane	2(3.45)
Choroidal Rupture	4(6.90)
Optic Disc Atrophy + Retinal Scar + Macular	1(1.72)
Scar	
Retinal Pigment Epithelium Atrophy	1(1.72)
Macular Scar	1(1.72)
Retinal Scar	1(1.72)
Epiretinal Membrane + Atrophied Retinal Hole	1(1.72)

## DISCUSSION

Ocular trauma is a major cause of visual impairment worldwide and is classified into open globe and closed globe injuries. Open globe injuries involve a full-thickness wound in the eye wall due to perforation, laceration, or rupture, while closed globe injuries affect an intact eye but can involve different parts of the eye.^9^ The pattern of closed globe injuries can vary greatly depending on geography, ethnicity, and occupation.

The average age of patients was 31.86±13.08 years, with a range of 18 to 71 years. This is consistent with a study by Sthapit et al.^7^ who reported a mean age of 28.43 years, and with a study by Kinderan et al., which found an average age of 28.3 years in a larger patient group. ^10^ The study results highlight that ocular trauma often affects younger individuals. The younger age predominance may be attributed to higher exposure to risky activities and occupations, such as manual labor or outdoor work, which are more common in this demographic, aligning with findings from Sthapit et al.who noted similar trends in Nepal.^7^

A male predominance was observed in our study, with 48 (82.76%) patients being male. This is in line with other studies. Sthapit et al. found 72.3% male patients, and Godar ST et al.found 66.5% male cases.^7,11^ Khatry et al. also reported a higher percentage of male patients (50.9%).^5^ This may be due to more males being involved in outdoor, high-risk jobs, increasing their chances of injury.

The ethnic distribution in our study reflected Nepal’s diverse population. Thirty-six (62.07%) patients were from the Indo-Aryan group while 22 (37.93%) were from the Tibeto-Burman group. Regarding occupation, 20 (34.48%) patients were students, 13 (22.41%) were in service, 11 (18.97%) were in business, 10 (17.24%) were in agriculture, and 4 (6.90%) were homemakers. Khatry et al. reported that farmers and domestic workers were most affected, and Sthapit et al. also found household areas to be the most common site of injury.^5,7^ The occupational diversity suggests that ocular trauma is not limited to specific professions but may be influenced by environmental factors and activity levels, with students possibly affected due to recreational risks, a pattern also noted in Khatry et al.^5^

Educational background showed that 32 (55.17%) patients had completed secondary education, 18 (31.03%) had higher education, and only 2 (3.45%) were illiterate. Hashemi et al. found the highest prevalence of ocular trauma was among illiterate people, which was significantly different compared to our study.^12^ This suggests that ocular trauma affects educated individuals as well. The most common cause of injury in our study were fist injuries in 19 (32.76%) patients followed by blunt trauma from wooden objects in 12 (20.69%) patients, fall injuries in 10 (17.24%) patients, and injuries due to stones in 5 (8.62%) patients. Sthapit et al. found wooden objects to be a common cause (19.6%), while Godar et al identified road traffic accidents as the leading cause.^7,11^

A total of 35 (60.34%) patients presented after 24 hours of injury, which may be due to poor access to healthcare, transportation problems, and lack of awareness, as also noted by Pradhan et al. who reported an average delay of 13.9 days following earthquake.^13^ The delay in presentation may reflect limited access to healthcare or lack of immediate symptom recognition, particularly in rural areas, which could explain the higher delayed presentation in our study compared to urban studies.^7^

In our study, the left eye was affected in 32 (55.17%) cases and the right eye in 26 (44.83%), with no bilateral cases. In a previous study conducted by Sthapit et al right eye was involved in 55 cases, left eye in 40 cases and both eyes in 17 cases, which was different from our study as no bilateral eye involvement and left eye was more involved than right eye.^7^ This shows there is no association between the ocular injury and the laterality of the eye. RAPD was seen in only 4 (6.90%) patients at the time of presentation, indicating possible traumatic optic nerve damage leading to poor final visual acuity. Pieramici et al. found that if RAPD was present, final visual acuity was significantly worse, which is similar to our study.^14^ At initial presentation, vision ranged from normal to blindness. About 30 (51.72%) had normal vision, while others had varying degrees of impairment, including 7 (12.07%) who were blind. After 6 months, 46 (79.31%) had normal vision, showing good recovery with treatment. The mean visual acuity improved significantly from 0.566 ± 0.652 logMAR at presentation to 0.222 ± 0.440 logMAR at six months (mean difference = 0.344 logMAR; 95% CI: 0.233-0.453). Pradhan et al.had a broader range of visual outcomes.^13^ The improvement in our patients may be due to timely treatment and regular follow-up.

Posterior segment findings included 27 (46.55%) patients with isolated commotio retinae, 5 (8.62%) with vitreous hemorrhage, and 2 (3.45%) with Berlin’s edema. Other complications like retinal detachment, retinal holes, choroidal rupture, and macular holes were less common. Studies by Phillips et al. and Williams et al. showed different patterns of posterior segment injuries depending on the type of trauma, such as blast injuries versus blunt force.^15,16^ In our study, the high rate of commotio retinae was likely due to blunt trauma, especially from fists, which is a frequent cause of closed globe injuries in physical assault and altercations.

Treatment was mostly medical, with 37 (63.79%) patients receiving topical steroid therapy. Nine (15.52%) patients received combined surgical and medical treatment, while 7 (12.07%) patients were observed without treatment. Only a few patients needed surgeries for serious injuries. At the 6-month follow-up, 44 (75.86%) had no complications. Choroidal rupture in 4 (6.90%) pateints and optic disc atrophy in 2 (3.45%) pateints were the most frequent complications. In the study conducted by Kadappu et al. repeat vitreous hemorrhage, secondary glaucoma, and cataract were seen after closed globe injury, which is different from our study.^17^ In the study conducted by Simanjuntak et al. on visual outcome following blunt trauma, the poor visual outcome was due to vitreous hemorrhage in 2 (2%) of 97 patients, and choroidal rupture in 1 (1%) patient.^18^ The result was different from our study due to the mode of injury, as the common cause of injury was a toy gun, and in our study, it was fist injury. The mean age in this study was 19.11 ± 12.75 years, which was different from our study. This signifies that the age group and mode of injury influence the clinical features and sequelae following injury to the eye.

Our study had some limitations. The follow-up period made it difficult to assess long-term outcomes. This was a single-center study; however, a multi-center study would be more beneficial, as it could include a broader and more diverse population, making the findings more representative of the general population. Other limitations include the small sample size and absence of blinding of investigators. Public health efforts should focus on raising awareness and improving access to early care to reduce the incidence of visual disability.

## CONCLUSIONS

Closed globe injuries are a notable cause of visual impairment in Nepal, predominantly affecting young males through blunt trauma. The study highlights the involvement of the posterior segment as a significant contributor to visual morbidity. While timely management yields favorable outcomes, delayed presentation remains a critical issue. Continued emphasis on early diagnosis and targeted management strategies may help minimize long-term complications in such cases.

## Data Availability

The data are available from the corresponding author upon reasonable request.
